# Supplementary material for: Applying interpretable machine learning to assess intraspecific trait divergence under landscape‐scale population differentiation
Source: Appl Plant Sci. 2025 Jun 18;13(3):e70015. doi: 10.1002/aps3.70015 (PMC12188625; doi:10.1002/aps3.70015)
Supplement: Supplementary file 1 — Appendix S1. Percentage of missing values for each functional trait in the full dataset. [file APS3-13-e70015-s001.pdf]

DOI 10.1002/aps3.70015

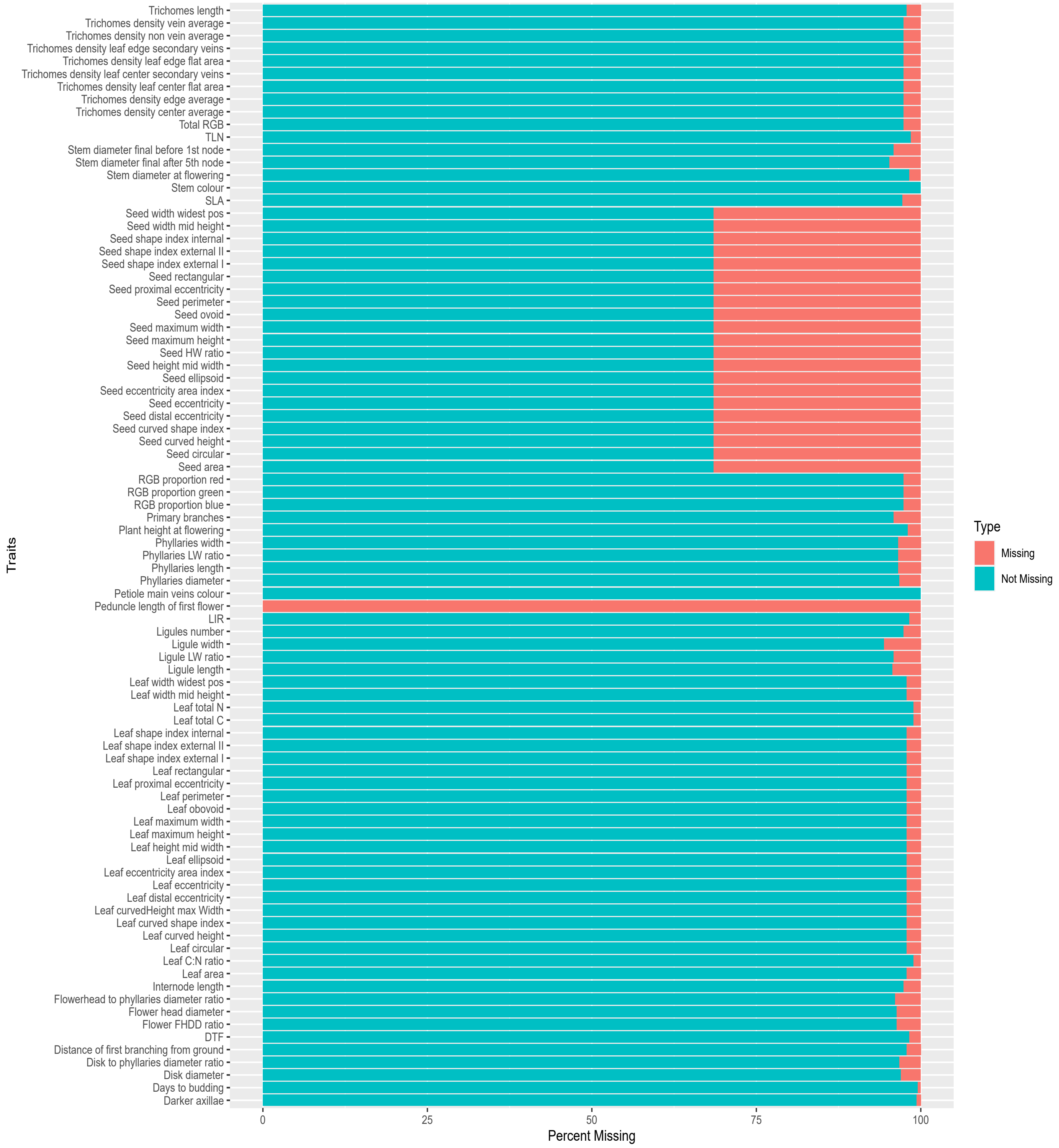

Appendix S1. Percentage of missing values for each functional trait in the full dataset.

DOI 10.1002/aps3.70015

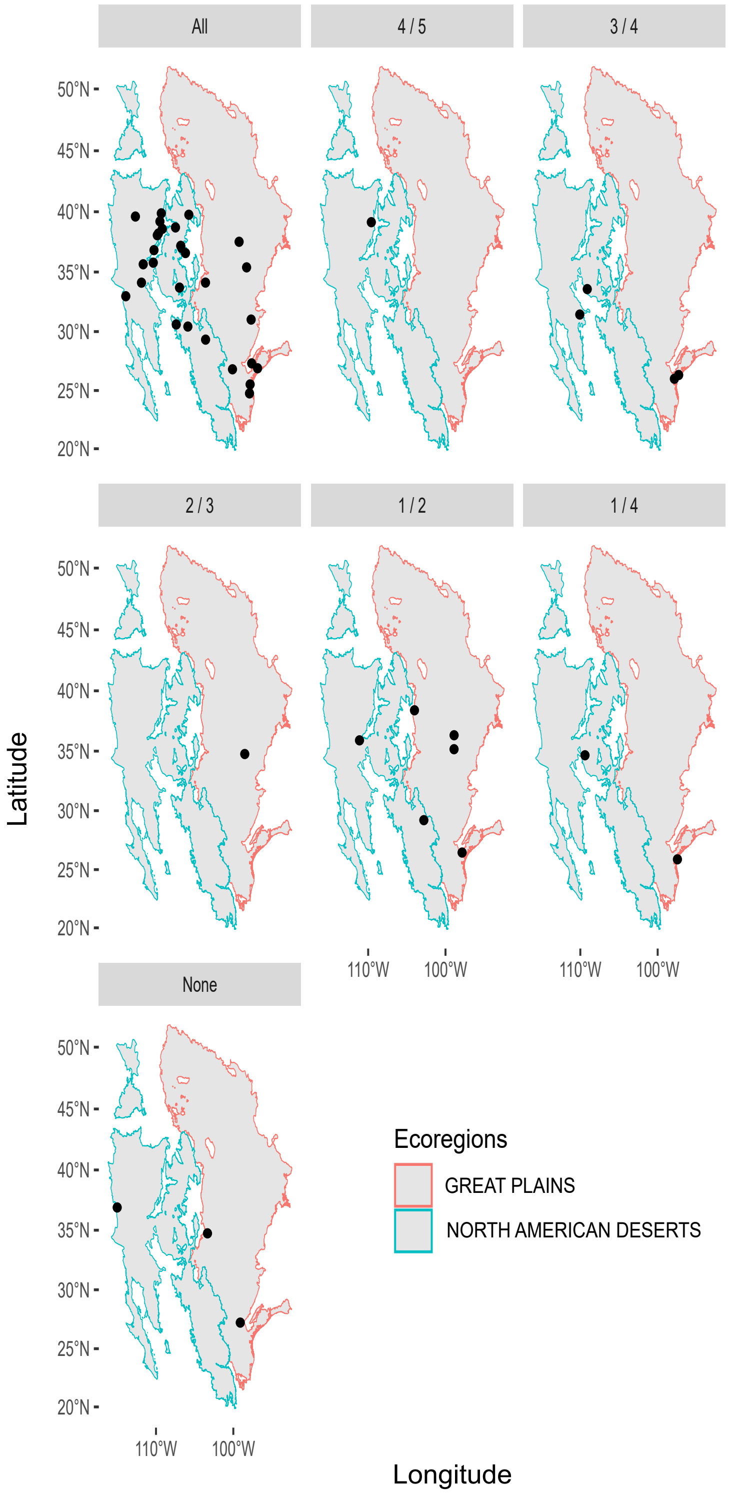

Appendix S8. Visualizations of the predictions made by the random forest (RF) classifier, where points represent populations of *Helianthus annuus*. Each facet represents populations where a given proportion of genotypes was correctly predicted, arranged in descending order – all genotypes correctly predicted, four out of five correctly predicted, three out of four correctly predicted, two out of three correctly predicted, one out of two correctly predicted, one out of four correctly predicted, or none correctly predicted.
